# Ferrous iron formation following the co-aggregation of ferric iron and the Alzheimer's disease peptide β-amyloid (1–42)

**DOI:** 10.1098/rsif.2014.0165

**Published:** 2014-06-06

**Authors:** J. Everett, E. Céspedes, L. R. Shelford, C. Exley, J. F. Collingwood, J. Dobson, G. van der Laan, C. A. Jenkins, E. Arenholz, N. D. Telling

**Affiliations:** 1 Institute for Science and Technology in Medicine, Keele University, Stoke-on-Trent, Staffordshire ST4 7QB, UK; 2 College of Engineering, Mathematics and Physical Sciences, University of Exeter, Exeter EX4 4QL, UK; 3 The Birchall Centre, Lennard-Jones Laboratories, Keele University, Staffordshire ST5 5BG, UK; 4 School of Engineering, University of Warwick, Coventry CV4 7AL, UK; 5 J. Crayton Pruitt Family Department of Biomedical Engineering, University of Florida, Gainesville, FL 32611, USA; 6 Department of Materials Science and Engineering, University of Florida, Gainesville, FL 32611, USA; 7 Magnetic Spectroscopy Group, Diamond Light Source, Didcot, Oxfordshire OX11 ODE, UK; 8 Advanced Light Source, Lawrence Berkeley National Laboratory, Berkeley, CA 94720, USA

**Keywords:** Alzheimer's disease, β-amyloid, wüstite, redox, X-ray absorption

## Abstract

For decades, a link between increased levels of iron and areas of Alzheimer's disease (AD) pathology has been recognized, including AD lesions comprised of the peptide β-amyloid (Aβ). Despite many observations of this association, the relationship between Aβ and iron is poorly understood. Using X-ray microspectroscopy, X-ray absorption spectroscopy, electron microscopy and spectrophotometric iron(II) quantification techniques, we examine the interaction between Aβ(1–42) and synthetic iron(III), reminiscent of ferric iron stores in the brain. We report Aβ to be capable of accumulating iron(III) within amyloid aggregates, with this process resulting in Aβ-mediated reduction of iron(III) to a redox-active iron(II) phase. Additionally, we show that the presence of aluminium increases the reductive capacity of Aβ, enabling the redox cycling of the iron. These results demonstrate the ability of Aβ to accumulate iron, offering an explanation for previously observed local increases in iron concentration associated with AD lesions. Furthermore, the ability of iron to form redox-active iron phases from ferric precursors provides an origin both for the redox-active iron previously witnessed in AD tissue, and the increased levels of oxidative stress characteristic of AD. These interactions between Aβ and iron deliver valuable insights into the process of AD progression, which may ultimately provide targets for disease therapies.

## 1. Introduction

Iron is fundamentally involved in multiple processes within the human brain, including myelin synthesis, neurotransmitter function, along with energy production made possible via its ability to change valence states [1,2]. It is this ability to change valence states however that can also lead to iron toxicity. Under normal circumstances, iron is stored as ferrihydrite, a redox-inactive ferric oxyhydroxide within the storage protein ferritin [3]. However, when ferritin function is compromised, or excess iron concentrations are reached, increased levels of redox-active labile iron form [4–7]. This labile iron is free to participate in the Fenton reaction resulting in the generation of reactive oxygen species (ROS) which go on to induce oxidative stress and neuronal damage [8–11].

Oxidative stress is also a key factor in Alzheimer's disease (AD), a fatal neurodegenerative disorder that is characterized by widespread and extensive neuronal death, resulting in memory loss, psychosis and dementias [5,12,13]. Although not fully understood, it is becoming accepted that the accumulation of the neurotoxic peptide β-amyloid (Aβ) within neurons is fundamental to the pathology of AD [14,15]. Aβ accumulation leads to its extracellular deposition, appearing as senile plaques, a hallmark lesion of the disease [16,17]. Aβ has also been shown to induce the formation of intracellular neurofibrillary tangles [18], and it is these manifestations of Aβ accumulation that result in a disruption of energy production, activation of the immune system and disturbances to normal neuronal function that ultimately result in cell death [19]. Additionally, Aβ has been shown to be capable of directly inducing the production of free radicals, thereby contributing to the oxidative stress characteristic of the AD brain [20,21].

Since Louis Goodman's case studies conducted in the 1950s, a link has been suggested between increased regional brain iron concentrations and areas of AD pathology [22]. Interestingly, increased levels of iron have been shown to exist in areas of Aβ accumulation such a senile plaques and neurofibrillary tangles, suggesting that Aβ may act as a sink for iron deposition [22–24]. Further investigations have identified increased amounts of redox-active iron(II) within AD tissue [25–32]. The presence of such redox-active iron would represent a substantial source of ROS production through the previously mentioned Fenton chemistry [28]. With the accumulation of iron within brain structures, and the occurrence of oxidative stress being recognized as early stage events in pathogenesis, the formation of redox-active iron may represent a key step in the development of the disease [33].

The origin of this redox-active iron is unclear, but it has been suggested that its formation may be a result of malfunction in the iron storage protein ferritin, or the interaction of Aβ with poorly liganded, or free iron forms [34]. Recent findings by Jiang *et al*. [35] indicate Aβ to be capable of binding to iron, and spectrophotometric studies by Khan *et al*. [36] have shown Aβ to be capable of reducing iron(III) to iron(II) phases in solution *in vitro.* Further to this, electron tomography studies have revealed the presence of redox-active iron within senile plaque material taken from the AD brain [29]. This evidence suggests that Aβ may act to bind natural ferric forms of iron, before chemically reducing them into pathological ferrous iron phases capable of inducing oxidative stress. In addition to iron, other metals such as aluminium, copper and zinc have been shown to accumulate in areas of AD pathology, with synergies between these metals possibly altering the mechanisms of Aβ/iron interaction [37,38].

Despite these observations, the relationship between Aβ and iron is poorly understood, and the products of Aβ/iron interaction remain unknown. Here, we use a combination of methods, including scanning transmission X-ray microscopy (STXM), X-ray absorption spectroscopy (XAS), X-ray magnetic circular dichroism (XMCD), transmission electron microscopy (TEM) and spectrophotometric iron(II) quantification, to examine the interaction between Aβ and synthetic ferric iron indicative of biological iron forms. Moreover, the effect of the addition of aluminium(III) upon these processes is assessed.

STXM and XAS are synchrotron-based techniques that allow the element-specific imaging of a given structure to a spatial resolution of 20 nm, and the determination of the oxidation state of –3*d* transition metals in a composite material, respectively. In this study, STXM was used to image the iron and amyloid content of structures formed following the incubation of Aβ with iron(III). Further to this, XAS was used to detect any changes in the oxidation state of iron when incubated with Aβ (either in the presence or the absence of aluminium) over a 144 h period. XMCD measurements were used to confirm the oxidation and magnetic state of the iron.

We report Aβ to be capable of incorporating and accumulating synthetic iron(III) into aggregate structures, with this interaction resulting in Aβ-mediated chemical reduction of iron(III) to a pure iron(II) phase. Iron(II) quantification assays confirmed the reduction of iron(III) by Aβ in suspension, while the addition of aluminium(III) was shown to enhance the reductive capacity of Aβ upon iron and also enabled iron redox cycling. Taken together these results offer an explanation for the increased iron levels witnessed in areas of AD pathology, and also suggest an origin for the redox-active iron forms and oxidative stress previously witnessed in AD tissue, thereby shedding light on the process of AD pathogenesis.

## 2. Material and methods

### 2.1. Scanning transmission X-ray microscopy and transmission electron microscopy

Element-specific images revealing the structure and composition of Aβ/iron(III) aggregates with a spatial resolution of approximately 20 nm were obtained by performing STXM on the PolLux beamline at the Swiss Light Source (Paul Scherrer Institute, Switzerland). TEM was performed using a JEOL 1230 microscope operating at 100 kV. Where both STXM and TEM were employed on the same sample membrane, the STXM measurements were performed first to exclude the effect of electron beam damage to the aggregates.

#### 2.1.1. Preparation of samples

Frozen Aβ(1–42) (Bachem) was thawed and dissolved in 0.1 M sodium hydroxide (NaOH) to create a 1 mg ml^–1^ (220 µM) stock. NaOH was used to dissolve any insoluble Aβ aggregates that may have formed during peptide storage, thereby reverting amyloid aggregation (as recommended by the peptide manufacturer). The Aβ stock was left at room temperature for 30 min to ensure complete peptide dissolution before being immediately added to modified Krebs–Henseleit (KH) buffer (pH 7.4; 100 mM PIPES). Two amyloid treatments were prepared. (i) To assess the co-aggregation of Aβ and iron, 18 mM iron(III) nitrate solution was added to KH buffer immediately after Aβ and the resulting Aβ/iron(III) hydroxide suspensions were left to incubate at 37°C for 96 h before sampling. (ii) In order to investigate the inclusion of iron into pre-formed Aβ structures, Aβ solutions in KH buffer were allowed to incubate for 48 h at 37°C before the addition of 18 mM iron(III) nitrate solution. Following the addition of iron(III), Aβ/iron(III) suspensions were allowed to incubate for a further 30 min before sampling. For both Aβ/iron preparations, final peptide and iron concentrations were 35 and 370 µM, respectively.

Small volumes (15 µl) of Aβ/iron(III) hydroxide suspensions were deposited onto silicon nitrate membranes (75 nm thickness, DuneSciences), and excess liquid removed with filter paper to prevent any artefacts arising due to the drying of the suspensions. The membranes used were pre-treated with a hydrophilic compound to encourage the deposition of Aβ/iron(III) structures onto the membrane windows. Following sample deposition, the silicon membranes were loaded onto aluminium plates for STXM examination.

#### 2.1.2. Scanning transmission X-ray microscopy analysis

Carbon maps revealing the amyloid structure of Aβ/iron aggregates were created by performing raster scans across Aβ/iron structures at the peak carbon *K*-edge energy (288 eV) and off-peak energy (282 eV). Scans at multiple energies across the carbon *K*-edge (280–320 eV) were performed across the Aβ/iron aggregates in order to determine the X-ray absorption spectrum characteristic of Aβ. Maps showing the iron content of Aβ structures were created by conducting scans at the iron *L*_3_ peak energy (710 eV) and off-peak energy (705 eV), with differences in these scans providing the location of any iron within the Aβ aggregates. As the absorbance energies of iron are higher than those of carbon, STXM carbon analysis of Aβ structures was conducted prior to analysis of the iron content, in order to minimize X-ray-induced damage to the amyloid structure.

### 2.2. X-ray absorption spectroscopy and iron(II) quantification in suspension

#### 2.2.1. Preparation of iron/amyloid suspensions

Iron(III) hydroxide suspensions were prepared by diluting 18 mM iron(III) nitrate (Sigma-Aldrich) in deionized water and subsequently neutralizing to pH 7 with 1 M NaOH, giving an iron concentration of 440 µM. Suspensions containing both 440 µM iron(III) and 440 µM aluminium(III) at pH 7 were created from 18 mM iron(III) nitrate, and 37 mM aluminium(III) nitrate (Perkin-Elmer) in a similar manner as described above, with aluminium(III) nitrate being added after the iron(III) nitrate. All suspensions were sonicated for 5 min prior to Aβ addition to encourage a homogeneous metal distribution.

Frozen Aβ(1–42) was thawed and dissolved in 0.1 M NaOH to create a 1 mg ml^−1^ (220 µM) Aβ stock. This Aβ stock was allowed to sit for 30 min to ensure complete peptide dissolution before being added to the previously prepared metal suspensions. Aβ/metal suspensions were again neutralized to pH 7 following the addition of the Aβ stock, via the addition of 0.1 M hydrochloric acid (HCl). Final Aβ and metal concentrations were 35 and 370 µM, respectively. Amyloid-free iron suspensions were created in the same manner as above with the substitution of deionized water in place of Aβ. All Aβ/iron suspensions and amyloid-free controls were incubated at 37°C over a period of 144 h.

#### 2.2.2. X-ray absorption and X-ray magnetic circular dichroism spectroscopy

Small volumes (15 µl) of the Aβ/metal suspensions and their Aβ-free controls were pipetted onto carbon/formvar-coated copper TEM grids (200 mesh; Agar Scientific), and excess liquid removed using filter paper. Sampling was performed after 30 min, 48 h and 144 h of metal incubation with Aβ. The grids were then mounted onto copper plates for X-ray absorption (XAS and XMCD) examination. These samples were kept under anoxic conditions throughout the experimental process to prevent any changes in iron valence chemistry (see the electronic supplementary material for more information regarding anoxic methodology).

XAS and XMCD measurements were conducted on beamline 4.0.2 at the Advanced Light Source (Berkeley Laboratory, USA) and beamline I10 at the Diamond Light Source (Oxfordshire, UK). Prior to spectra acquisition, two-dimensional maps at a spatial resolution of 100 µm revealing areas of iron accumulation within the sample area were created by raster scanning across the sample grid at the iron *L*_3_ absorption peak energy (710 eV) and off-peak energy (705 eV; see the electronic supplementary material, figure S1). Differences in these maps revealed areas containing substantial iron deposition. Detailed XAS/XMCD scans were then performed on these areas of iron accumulation across the entire iron *L*_2,3_ absorption edge (700–740 eV), providing information regarding both the oxidation state and the magnetic properties of the iron. However, not all iron deposits located in this way provided a sufficiently stable signal for full XAS/XMCD analysis to be performed.

The X-ray absorption (XAS) spectra, revealing the oxidation state of iron in the samples, were recorded using the total electron yield method, while the magnetic properties were probed by analysing XMCD spectra. The latter were obtained by measuring the difference in X-ray absorption using circularly polarized X-rays, when a 0.6 T magnetic field was applied in opposing orientations along the X-ray beam direction.

### 2.3. Iron(II) quantification in suspension: Ferrozine assay

Spectrophotometric determination of the iron(II) content of Aβ/iron(III) hydroxide suspensions was achieved by performing a Ferrozine iron(II) colorimetric quantification assay. Ferrozine is a compound that selectively binds to iron(II) ions in solution/suspension, causing the formation of a stable magenta complex that absorbs light at a wavelength of 562 nm [39]. The degree of this colour change is directly correlated to the amount of iron(II) present upon Ferrozine addition, and therefore can be used to assess the iron(II) content of a given solution/suspension.

To assess the iron(II) content of the Aβ/metal suspensions, small volumes of sample were removed and digested in 0.5 M HCl for 3 h at room temperature to release any bound iron from Aβ structures in order to enable Ferrozine binding. Acid-digested samples were then added to 2 mM Ferrozine, and absorbance read at 562 nm. The total iron content of the Aβ/metal suspensions was recorded by adding small volumes of the sample solution to 0.5 M HCl and 6.25 M hydroxylamine hydrochloride (an iron-reducing agent), at room temperature for 3 h. These reduced samples were then added to 2 mM Ferrozine and absorbance read at 562 nm as before. From these measurements, the iron(II) content as a percentage of total iron content was determined. The iron(II) contents of Aβ-free iron controls were assessed in the same way to provide iron(II) background levels for all iron suspensions used.

Spectrophotometric measurements were performed as described above on samples taken after 0, 24, 48, 72, 120 and 144 h of metal incubation with Aβ. No iron(II) quantification data were collected after 96 h of incubation owing to the limited amount of sample volume available.

### 2.4. Statistical analysis

Statistical analysis of the data obtained from iron(II) quantification in suspension was performed using a one-way analysis of variance (GraphPad Prism 6). This is a method of comparing sample means for two or more populations. The null hypothesis of equal means was rejected at the 5% confidence level.

## 3. Results

### 3.1. The co-aggregation of iron and Aβ(1–42)

**Figure 1.**
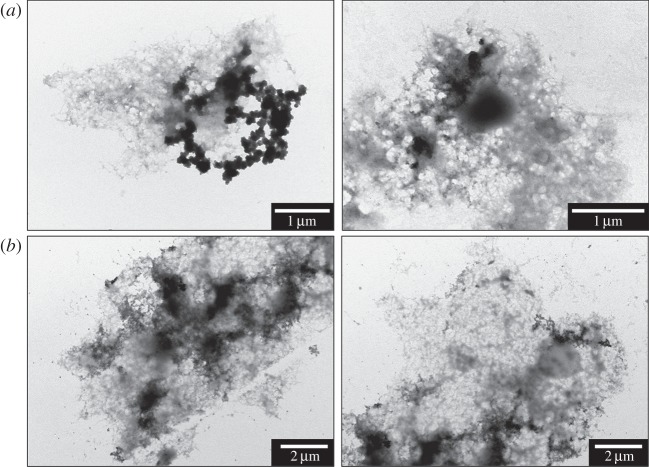
TEM images of typical Aβ structures formed following (*a*) 0.5 and (*b*) 96 h of Aβ incubation with iron(III).

TEM examination of amyloid structures incubated with iron(III) revealed the formation of fibrillar aggregates ranging from 1 to 50 µm in size and containing electron dense regions, typical examples of which are shown in figure 1. No obvious correlation between incubation time and aggregate size could be determined in these samples with both smaller (less than 5 μm) aggregates, and thick electron opaque regions (not shown) seen in many of the samples. In addition, it was observed that dense precipitates could be found in aggregates after only 30 min incubation (figure 1).

**Figure 2.**
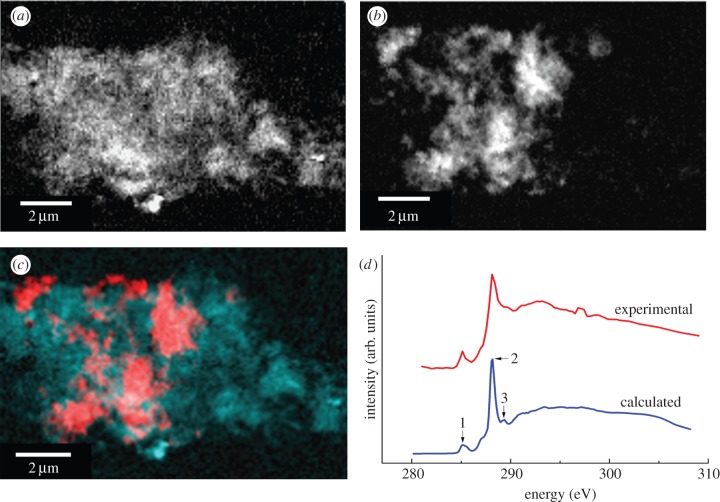
STXM images and carbon *K*-edge spectra of an Aβ/iron aggregate formed following 96 h of Aβ/iron(III) incubation. (*a*) Carbon map showing the Aβ structure of the aggregate. (*b*) Iron map revealing the iron content of the aggregate. (*c*) Carbon/iron composite image displaying both the Aβ (cyan) and iron (red) content shown in (*a*) and (*b*), respectively. (*d*) Calculated (blue) and experimental (red) carbon *K*-edge spectra for Aβ. The calculated spectrum was obtained using the procedures described by Stewart-Ornstein *et al.* [40].

To investigate the nature of the electron dense regions in the aggregates, STXM was performed. Where Aβ and iron(III) were added simultaneously and allowed to incubate for 96 h, carbon *K-*edge mapping of aggregates showed evidence of fine structure similar to that observed by TEM, together with dense carbon containing regions (figure 2*a*).

**Figure 3.**
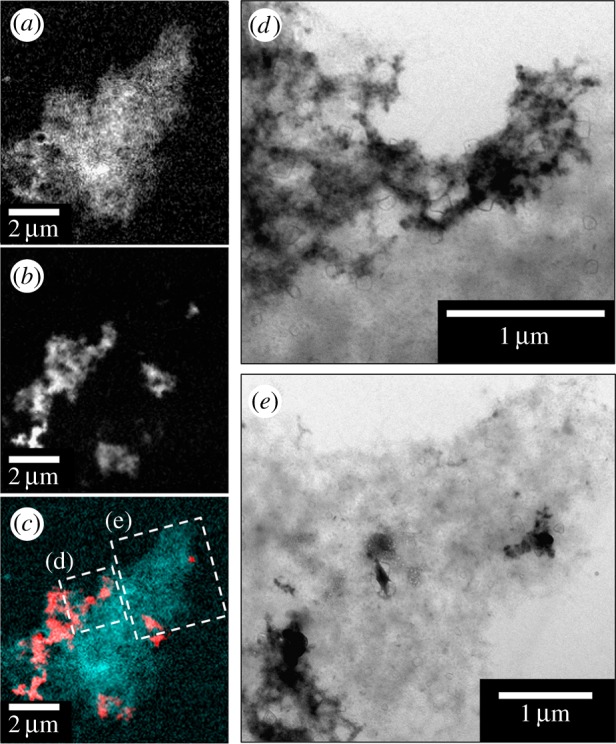
STXM and TEM images of an Aβ/iron aggregate formed following 96 h of Aβ/iron(III) incubation. (*a*) Carbon map showing the Aβ structure of the aggregate. (*b*) Iron map displaying the iron content of the same aggregate. (*c*) Carbon/iron composite image displaying both the Aβ (cyan) and iron (red) content shown in (*a*) and (*b*), respectively. (*d,e*) TEM images of the Aβ aggregate as labelled in (*c*).

Examination of the X-ray absorption by the aggregates across the entire carbon *K-*edge (280–320 eV) revealed spectra consistent with the calculated carbon spectrum for the amino acid sequence of the Aβ(1–42) peptide, confirming the amyloid content of the aggregates (figure 2*d*). Characteristic features of the peptide spectrum are a low energy peak (labelled 1, figure 2*d*) which arises from the aromatic amino acids, the dominant π* amide peak (labelled 2) and a weaker shoulder feature (labelled 3) associated with arginine [40]. Iron *L-*edge examination of the same aggregates showed regions of iron accumulation within the amyloid structure, indicating the co-aggregation of iron with Aβ (figure 2*b*). Iron was found to possess a fine structure similar to the amyloid, and regions of dense iron were contained within the aggregates (figure 2*c*). A comparison of aggregates measured by both STXM and TEM showed that many of the dense regions seen by TEM were owing to accumulated iron within the aggregate (figure 3). However, no evidence of similar iron accumulation was observed in the absence of Aβ.

**Figure 4.**
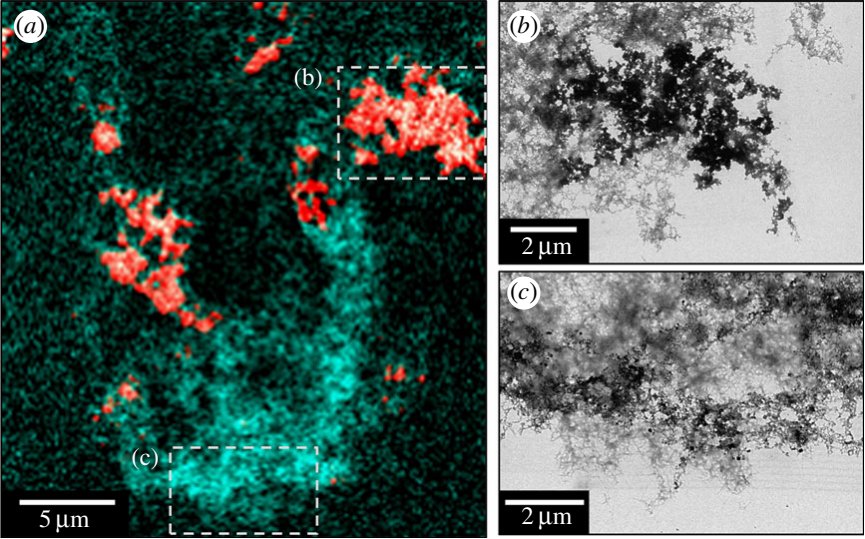
STXM and TEM images of a Aβ aggregate allowed to incubate for 48 h prior to the addition of iron(III). (*a*) A composite image showing the Aβ (cyan) and iron (red) content of the Aβ/iron aggregate. TEM images showing the fibrillar structure of the Aβ/iron aggregate in areas of high (*b*) and low (*c*) iron content as displayed in (*a*).

Carbon *K-*edge and iron *L-*edge examination of Aβ samples allowed to incubate in KH buffer for 48 h prior to the addition of iron(III) led to the observation of Aβ structures indistinguishable from those formed where Aβ and iron(III) were added simultaneously (figure 4*a*). Combined STXM and TEM images of these aggregates showed them to be fibrillar in nature containing multiple areas of iron accumulation (figure 4), suggesting iron had incorporated into Aβ structures that had formed prior to the addition of the metal.

### 3.2. Oxidation state of iron, following Aβ interaction

#### 3.2.1 Iron(III) series

**Figure 5.**
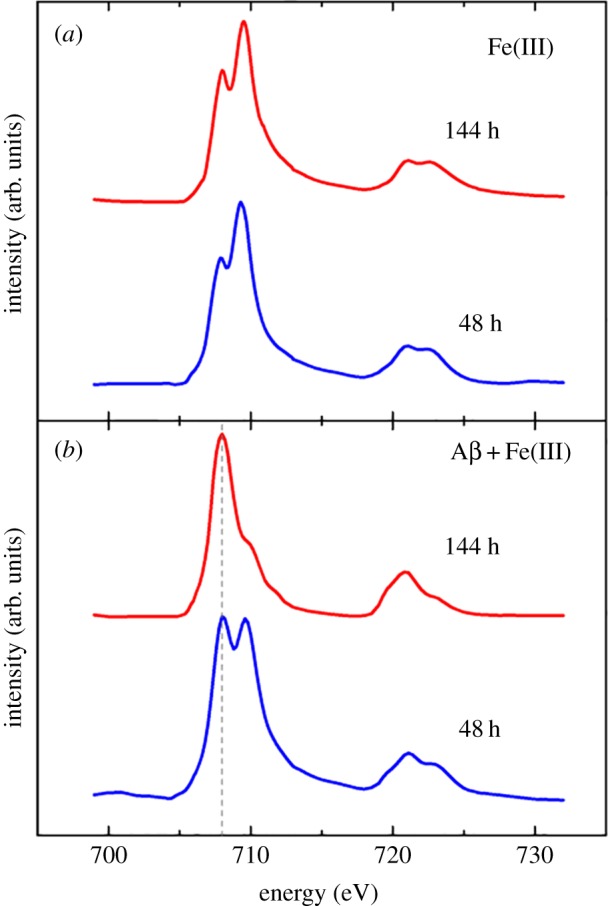
Iron *L-*edge X-ray absorption spectra of iron(III) in the absence (*a*) and presence (*b*) of Aβ after 48 and 144 h of incubation. Grey dashed line at 708 eV in (*b*) provides a visual guide for iron(II) content. (Online version in colour.)

**Figure 6.**
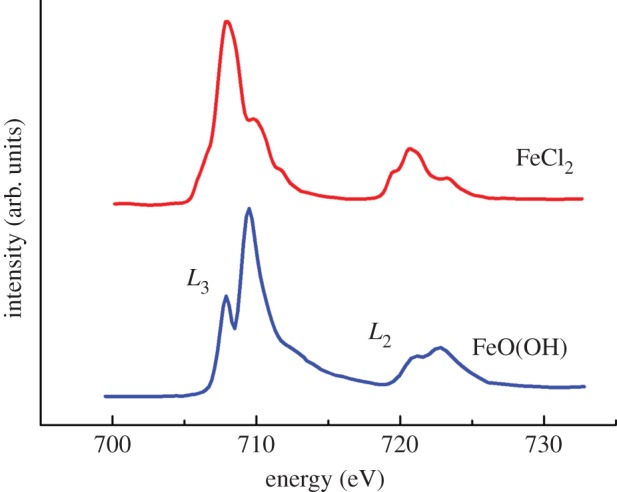
Reference iron *L*-edge X-ray absorption spectra for iron(III) (FeO(OH)) and iron(II) (FeCl_2_). The iron *L*_2_ and *L*_3_ regions are labelled for FeO(OH). (Online version in colour.)

Iron *L*_2,3_ X-ray absorption spectra obtained from iron(III) samples maintained under anoxic conditions both in the absence and the presence of Aβ are shown as a function of time in figure 5. Pure iron(III) minerals provide X-ray absorption spectra comprised of a low energy *L*_3_ shoulder feature at 708 eV, followed by a dominant peak at 709.5 eV arising from the presence of Fe^3+^ cations (see, for example, the reference iron(III) (FeO(OH)) spectrum in figure 6). As the low energy spectral feature of iron(III) minerals is located at the same energy point (708 eV) as the peak from Fe^2+^ cations, increases in the iron(II) content of a given iron mineral appear to cause an enhancement in this 708 eV feature.

Where iron(III) was incubated in the absence of Aβ, the XAS spectra obtained were seen to resemble that of iron(III) references, but with slightly enhanced Fe^2+^ features (figure 5*a*). As the iron forms used for this experiment were amorphous in nature, this enhancement in iron(II) content is believed to be a result of X-ray beam exposure (in contrast to more crystalline iron forms such as magnetite which are stable under X-ray beam exposure). However despite this initial X-ray beam reduction, further X-ray exposure was not sufficient to form a pure iron(II) phase (see the electronic supplementary material, figure S2).

Where Aβ was incubated with iron(III) for 30 min, it was not possible to obtain a sufficient signal for reliable XAS and XMCD measurements. However, following 48 h of incubation with Aβ, iron was found to be in a predominantly Fe(III) phase (figure 5*b*), although clear evidence of enhanced Fe^2+^ cation features were apparent at 708 eV. This moderate reduction effect is again believed to be a result of X-ray beam exposure, as progressive reduction was seen with increasing periods of beam exposure (see the electronic supplementary material for an example of consecutive XAS measurements of an unstable iron form).

After 144 h of Aβ/iron incubation, iron was found to be reduced to a pure iron(II) phase (figure 5*b*) with a Fe^2+^ cation dominated XAS spectrum (for reference iron(II) spectra, see iron chloride (FeCl_2_) spectrum in figure 6). The Fe^2+^ cation peak at 708 eV is seen to be dominant, with the Fe^3+^ features at 709.5 eV having disappeared. This reduction effect was mirrored at the iron *L*_2_-edge (720–725 eV). As such pure iron(II) phases could not be formed in the absence of Aβ, the occurrence of this iron(II) mineral appears to be as a result of Aβ interaction with iron(III).

#### 3.2.2. Iron(III) and aluminium(III) series

**Figure 7.**
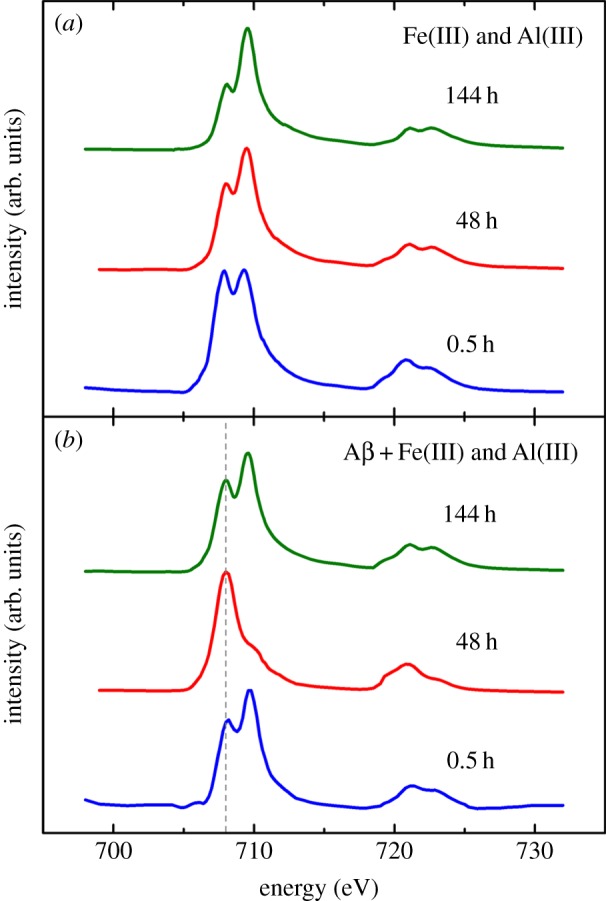
Iron *L-*edge X-ray absorption spectra for iron(III) aggregates containing aluminium(III) in the absence (*a*) and the presence (*b*) of Aβ. Incubation times are indicated above spectra. The grey dashed line at 708 eV in (*b*) is a visual indicator for iron(II) content. (Online version in colour.)

Iron *L*_2,3_ X-ray absorption spectra obtained from iron(III) hydroxide suspensions when incubated with aluminium(III) in the presence and the absence of Aβ are shown in figure 7. In the absence of Aβ, no evidence of a pure iron(II) mineral was found at any of the time points examined (figure 7*a*). Iron regions located after 144 h of incubation (figure 7*a*) show an iron *L*-edge spectra characteristic of a pure iron(III) mineral. However, reduced iron can be seen in the Aβ-free control sample after 30 min of incubation, manifesting as an enhancement in the shoulder at 708 eV. As for previously described results, this increase in Fe^2+^ cation peak intensity is thought to be owing to reduction caused by exposure to the X-ray beam. Despite this initial X-ray-mediated reduction, extensive periods of X-ray beam exposure did not lead to the formation of a pure iron(II) phase.

After 48 h of iron(III) incubation with Aβ and aluminium(III), iron *L*_2,3_ X-ray absorption spectra of Aβ/iron aggregates (figure 7*b*) resembled that of a pure iron(II) phase (figure 6). The *L*_3_ iron(II) peak at 708 eV had become dominant with the *L*_3_ iron(III) peak at 709.5 eV having disappeared. This pure iron(II) form was very similar to that seen after 144 h of Aβ/iron incubation in the absence of aluminium (figure 5*b*), indicating that a similar reduced iron phase had been formed but over a shorter incubation time. However, this pure iron(II) phase was not maintained after 144 h incubation, with iron reverting back to a largely iron(III) phase with some evidence of a Fe^2+^ cation content (figure 7*b*). This subsequent oxidation of the pure iron(II) phase for longer incubation times may indicate the establishment of an iron redox cycle.

### 3.3. Oxidative state of iron in suspension following Aβ interaction

**Figure 8.**
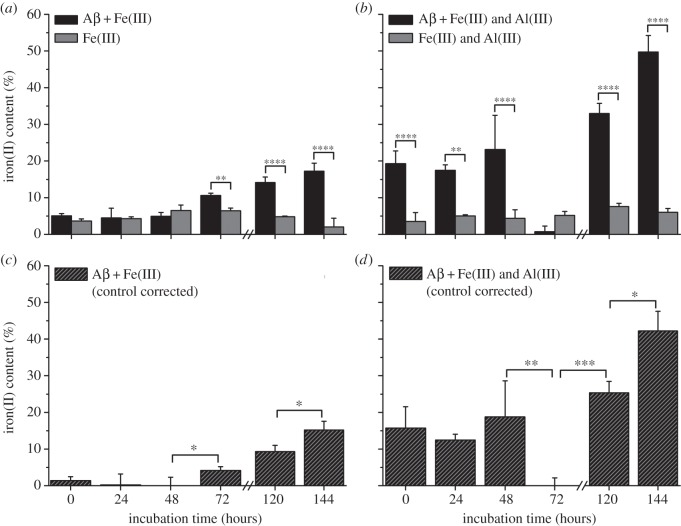
Spectrophotometric iron(II) quantification of Aβ suspensions containing iron(III) (*a,c*) and iron(III) and aluminium(III) (*b,d*). Iron(II) values as a percentage of total iron are shown for both Aβ/iron series and their Aβ-free controls in (*a,b*). Control corrected iron(II) values of Aβ/iron suspensions as a percentage of total iron are shown in (*c,d*). Note that measurements were not performed at 96 h. Error bars show standard deviation (*n* = 3); statistically significant differences in mean group values (by one-way ANOVA) are indicated at the following levels: *p < 0.05, **p < 0.01, ***p < 0.001, ^#^*p* < 0.0001.

To further investigate the reduction of iron(III) by Aβ in suspension, a spectrophotometric iron(II) quantification assay was performed. The iron(II) contents of suspensions containing Aβ and iron(III); Aβ, iron(III) and aluminium(III); and their Aβ-free controls are shown in figure 8*a,b*, and respective control corrected iron(II) contents are shown in figure 8*c,d*.

The iron(II) content of all Aβ-free suspensions was found to remain consistently low throughout all time points examined (figure 8*a,b*) with no clear evidence of iron(II) reduction being apparent. Where iron(III) was incubated with Aβ, no significant increases in control corrected iron(II) content were seen at the 0, 24 or 48 h time points (figure 8*c*). After 72 h, iron(II) content had risen to 4% and continued to rise to 9% at the 120 h time point and 15% after 144 h. Although iron(III) reduction by Aβ is evidenced, redox cycling was not apparent within this time frame, an observation consistent with X-ray absorption measurements (figure 5*b*).

In iron(III) suspensions containing Aβ and aluminium(III) an immediate conversion of iron(III) to iron(II) was observed, with control corrected iron(II) content accounting for 16% of the total iron content at time zero (figure 8*d*). Iron(II) levels then dropped to 12% after 24 h, before rising to 19% after 48 h incubation. This cycling of iron(II) continued, with iron(II) content disappearing entirely after 72 h incubation, before increasing to 25% and 42% of total iron content after 120 and 144 h, respectively.

These results show the reduction of iron(III) by Aβ in suspension, a result consistent with data collected via XAS (figures 5 and 7). The addition of aluminium appears to have a catalytic effect on iron reduction by Aβ, while also acting to increase the reductive capacity of Aβ, enabling the redox cycling of iron.

### 3.4. Magnetic state of iron following Aβ interaction

**Figure 9.**
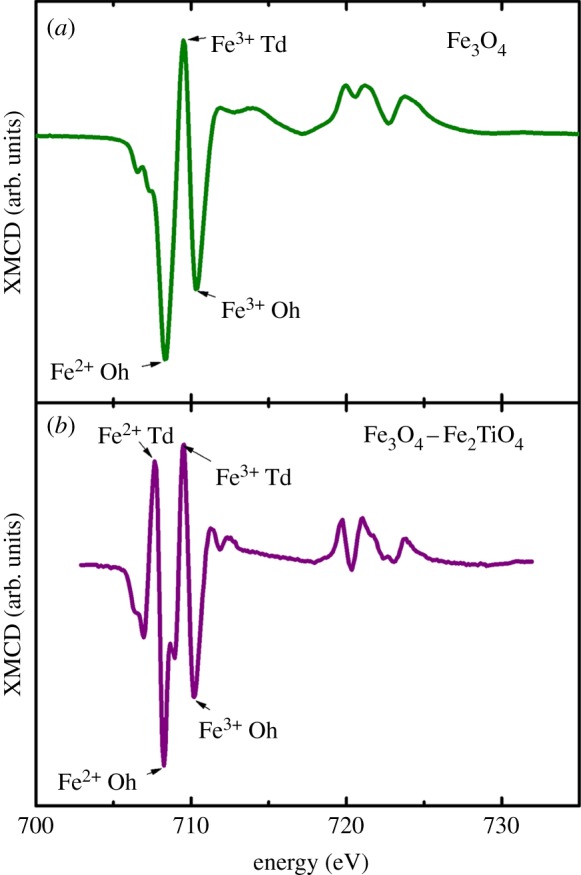
Iron *L-*edge XMCD reference spectra for (*a*) magnetite (Fe_3_O_4_) and (*b*) titanomagnetite (Fe_3_O_4_–Fe_2_TiO_4_). Tetrahedral (Td) and octahedral (Oh) crystal sites are labelled [41]. (Online version in colour.)

XMCD measurements were conducted across the iron *L*_2,3_ absorption edges of the samples in order to examine the magnetic state of the material present. Magnetic iron oxides such as magnetite (Fe_3_O_4_) generate a strong XMCD effect of 10–15%. This XMCD profile (figure 9*a*) appears as three peaks across the iron *L*_3_ region as a result of Fe^2+^ and Fe^3+^ cations occupying tetrahedral and octahedral crystal sites [42]. For titanomagnetite (figure 9*b*), an additional low energy positive peak is observed corresponding to Fe^2+^ cations occupying tetrahedral crystal sites [41]. The oxidation state of the mineral determines the relative intensities of these peaks, with oxidation causing an increase in Fe^3+^ cation intensity with respect to the Fe^2+^ cation peaks, and reduction causing an increase in the Fe^2+^ cation component with respect to the Fe^3+^.

**Figure 10.**
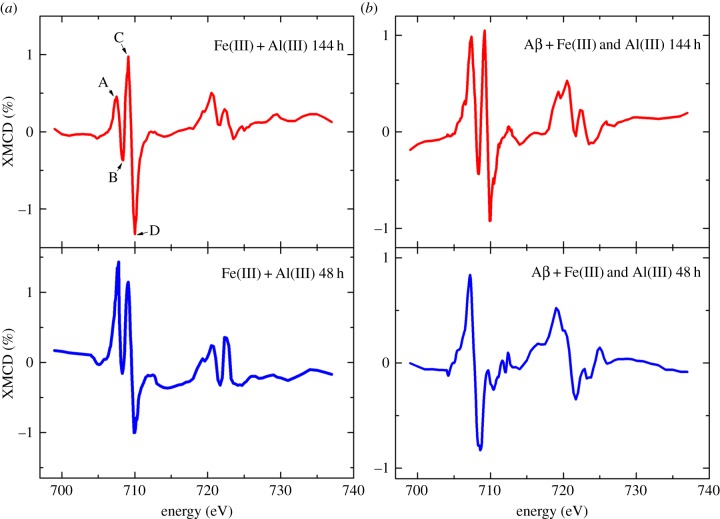
Iron *L-*edge XMCD spectra of iron(III) and aluminium(III) aggregates in the absence (*a*) and presence (*b*) of Aβ after 48 and 144 h of incubation. (Online version in colour.)

**Figure 11.**
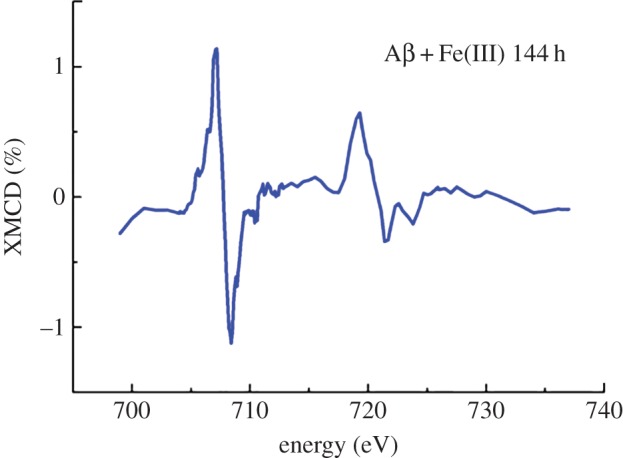
Iron *L*-edge XMCD spectrum of iron(III) with Aβ after 144 h of incubation. (Online version in colour.)

Iron *L*_2,3_ absorption-edge XMCD examination of iron(III) and aluminium(III) either in the presence or the absence of Aβ produced spectra with no evidence of strongly magnetic material. Instead a weak magnetic signal of 1–1.5% was observed throughout all samples examined (figures 10 and 11). Aβ-free iron(III) and aluminium(III) samples produced spectra comprised of two positive and two negative peaks (figure 10*a* (red); peaks A–D). By comparison with XMCD spectra obtained from titanomagnetite [41], these peaks appear to arise from the presence of both Fe^2+^ cations (figure 9*b*, peaks A and B) and Fe^3+^ cations (peaks C and D) that occupy tetrahedral and octahedral crystal sites.

The relative intensities of the peaks A–D shown in figure 9 reflect the oxidation state of magnetic Fe cations. XMCD examination of the pure iron(II) phase formed after 48 h of Aβ incubation with iron(III) and aluminium(III) showed dramatic enhancement of the Fe^2+^ cation features, with no evidence of Fe^3+^ cations on either the tetrahedral or octahedral sites being apparent (figure 10*b*). This XMCD signal is consistent with the formation of a weakly magnetic pure iron(II) phase. Subsequent oxidation after 144 h incubation can be seen as an increase in the Fe^3+^ cation XMCD features (figure 10*b*), mirroring the behaviour seen in the XAS spectra (figure 7*b*).

XMCD spectra obtained from the pure iron(II) phase formed following 144 h of incubation of Aβ with iron(III) (figure 11) were identical to those measured from Aβ incubations containing both iron(III) and aluminium(III) (figure 10*b*), suggesting a similar weakly magnetic iron(II) phase to have been formed, but over a longer period of time.

It should be noted that some distortions to the XMCD peak intensities are apparent owing to background X-ray absorption drift, resulting in a negative or positive slope across the XMCD profile (figure 10*a* (blue) and *b* (red)). However, despite these distortions, the overall trend of the XMCD spectra remains correct.

## 4. Discussion

Through the use of multiple techniques, including STXM, electron microscopy, XAS and spectrophotometric iron(II) quantification, it was found that the AD peptide Aβ(1–42) is capable of incorporating iron(III) minerals into fibrillar aggregate structures *in vitro*, with this interaction leading to the chemical reduction of iron(III) into a pure iron(II) phase.

Microspectroscopy (STXM) and TEM images of structures formed following the incubation of Aβ with iron(III) revealed the presence of large fibrillar amyloid aggregates containing multiple areas of iron accumulation. As amyloid and iron morphology were found to be closely correlated and iron was only observed when co-precipitated with Aβ, these data suggest that Aβ acts to accumulate iron within its structure with possible binding of iron occurring almost immediately (after 30 min incubation). This effect did not appear to be dependent upon the aggregation state of amyloid, with iron accumulation within Aβ aggregates occurring where iron(III) was added to pre-formed amyloid aggregates. These findings are in agreement with those published by Jiang *et al*. [35], who suggested Aβ to act as a metalloprotein capable of binding to iron.

The iron accumulation into amyloid aggregates that we have observed here could also provide an explanation for the increased concentrations of iron previously witnessed in AD plaque material [24]. With iron levels being shown to be increased in the AD brain [4,10,22,43], it is entirely feasible that Aβ acts to draw iron into its structure, where it then binds to the metal. Such a process would explain observations made by Lovell *et al.* [24], who used micro particle-induced X-ray emission analysis to show increased iron levels to exist within senile plaque material compared with surrounding brain tissue, and by Meadowcroft *et al.* [44], who used magnetic resonance imaging to show the accumulation of iron within Aβ plaques. Further to this, the ability of iron to bind to amyloid provides an explanation for the increased iron levels corresponding to areas of AD pathology as witnessed in mice overexpressing Aβ [45].

XAS examination of Aβ/iron(III) precipitates revealed the formation of a pure iron(II) mineral following prolonged periods (144 h) of Aβ/iron interaction. Such data show Aβ to be capable of directly reducing synthetic iron(III) in the absence of any other influencing factors. Further evidence of iron reduction by Aβ was provided via iron(II) quantification in suspension, where 15% of the total iron content was found to be in an iron(II) state when incubated following 144 h of Aβ/iron incubation. These results are consistent with our previous work where we found Aβ induced the reduction of the iron(III) oxyhydroxide mineral ferrihydrite [46]. The formation of pure iron(II) forms suggests Aβ to possess a strong reducing capacity upon iron, a result consistent with Khan *et al.* [36], who showed Aβ-mediated iron(III) reduction via spectrophotometric methods. These observations, combined with the previously stated STXM and TEM findings, indicate that Aβ acts to accumulate iron within its fibril structure with this interaction (binding) leading to Aβ-mediated iron reduction following prolonged periods of contact. This Aβ-induced iron reduction is expected to result in the oxidation of Aβ and the generation of ROS as described by Huang *et al*. [11]. However, limitations of the methodology used here prevented these reaction mechanisms from being confirmed in this study.

XAS examination of Aβ aggregates containing both iron(III) and aluminium(III) led to the observation of a similar pure iron(II) phase, but formed over a shorter interaction time (48 h) than where aluminium was absent. This catalytic effect of aluminium upon Aβ iron reduction was confirmed by iron(II) quantification assay, with the addition of aluminium leading to higher levels of iron reduction. Both XAS and iron(II) quantification assays revealed evidence of iron redox cycling where aluminium(III) was added to Aβ/iron(III) incubations, whereas no evidence of redox cycling was seen in its absence. These results show aluminium to act as an effective catalyst for the interaction of iron with Aβ, enabling the redox cycling of iron over the time period examined. These findings are also consistent with the work of Khan *et al*. [36], who show Aβ to be capable of inducing the redox cycling of iron, with the presence of aluminium(III) appearing to potentiate the reduction of iron(III) to iron(II); and also recent investigations by Ruiperez *et al.* [47], who show aluminium to promote the Fenton reaction by aiding the reduction of iron(III) to iron(II) [47].

The ability of Aβ to form iron(II) phases from iron precursors reminiscent of naturally occurring ferric iron provides a possible origin for the redox-active iron forms previously seen within AD tissue, such as the increased levels of the iron(II)-rich minerals magnetite and wüstite witnessed in pathological ferritin cores by Quintana *et al.* [27], along with the accumulation of magnetite-like material within AD plaque cores as observed by Collingwood *et al.* [29]*.* However, in contrast to our previous study on the interaction of Aβ with ferrihydrite [46], here we found no clear evidence for the formation of crystalline iron minerals as indicated by electron diffraction patterns.

XMCD analysis of the magnetic state of iron in samples prepared from both amyloid incubations and amyloid-free controls revealed iron cations in different crystal symmetry sites reminiscent of octahedral and tetrahedral coordination. This result was also found previously for ferrihydrite iron deposits [46] and thus appears to be a common feature of amorphous or nanocrystalline iron oxyhdroxides. The pure iron(II) phases observed by XAS, formed both with and without aluminium, were found by XMCD to contain Fe^2+^ cations arranged with opposing magnetic orientations. This implies the presence of antiferromagnetic coupling between Fe^2+^ cations on different crystal symmetry sites. These pure iron(II) phases could therefore represent an amorphous precursor for an antiferromagnetic iron(II) mineral such as wüstite.

## 5. Conclusion

From this study, it is apparent that Aβ is capable of interacting with iron in a manner that leads to the accumulation and co-aggregation of iron within Aβ structures, resulting in the chemical reduction of redox-inactive ferric iron to a redox-active ferrous iron form. With iron being abundant throughout brain tissues [1], and iron being shown to be increased in areas of AD pathology (in both human post-mortem tissue and AD transgenic models) [22,24,45], the ability of Aβ to induce the formation of redox-active iron(II) minerals from ferric precursors would represent a significant and sustained source of ROS capable of inducing widespread neuronal damage. The interaction of Aβ with iron could thus be an important contributor to the oxidative stress characteristic of AD, thereby playing a key role in the pathogenesis of the disorder. Furthermore, the apparent ability of Aβ to reduce iron(III) to an iron(II) phase even when iron is present in a 10 : 1 excess of Aβ strongly suggests that Aβ is an efficient biological iron-reducing agent. The catalytic function of aluminium(III) upon Aβ-mediated iron reduction as witnessed in this study is also of vital importance. With aluminium being implicated as a promoting factor for the development of AD, any synergies existing between Aβ, iron and aluminium are likely to influence the nature of AD progression [48]. Interestingly, redox-active iron has also been identified within rat microglia following kainate-induced neuronal injury. These findings indicate towards amyloid-independent sources of ferrous iron in the degenerating brain, which could further contribute to iron-induced toxicity within AD tissues [49].

As pure iron(II) phases do not occur naturally [27,50], these pathological iron biominerals may represent a target for future therapies. Removal of such forms of iron, or disruption in the ability of Aβ to interact with iron, may result in a reduction of the free radical burden associated with the AD brain and consequently a slowing of disease progression. The addition of aluminium appears to impact the reductive capacity of Aβ by increasing its ability to reduce iron(III) in suspension. Aluminium is also not naturally found within human tissues [51], and therefore its removal from brain tissue may act to reduce Aβ neurotoxicity, while not impacting healthy brain functions. In summary, key insights into the relationship between Aβ and iron have been made that provide valuable insights into the role played by iron in AD pathology.
